# Experiences of adolescents living with HIV on transitioning from pediatric to adult HIV care in low and middle-income countries: A Qualitative Evidence Synthesis Protocol

**DOI:** 10.1371/journal.pone.0296184

**Published:** 2024-02-05

**Authors:** Charné Petinger, Talitha Crowley, Brian van Wyk

**Affiliations:** 1 School of Public Health, University of the Western Cape, Cape Town, South Africa; 2 School of Nursing, University of the Western Cape, Cape Town, South Africa; International AIDS Vaccine Initiative, UNITED STATES

## Abstract

In South Africa, it is estimated that approximately 320,000 adolescents living with HIV (ALHIV) will transition from pediatric to adult antiretroviral treatment (ART) by 2028. However, the age period of 10–19 years is accompanied by a myriad of barriers that challenge the transition process, and continued adherence to ART. The transition process involves ALHIV taking charge of their own health and disease management which raises challenges for their retention in care. Managing transition becomes particularly challenging in low-resource contexts as their healthcare systems are not adapted to the specific needs it requires. There is a need to garner an understanding of existing transition practices which address the specific needs of adolescents and is optimized to their requirements and available resources within a low- or middle-income country context. This review will include all qualitative and mixed method studies which will facilitate a deeper understanding the experiences of ALHIV on transition experiences. The review will specifically look at studies conducted in low- and middle-income countries. The included studies must be presented in the English language and published between 2010–2023. The search strategy will be finalized with consultation with an information specialist. All three reviewers will be present throughout all stages of the review. One reviewer will work independently on the initial screening of studies and another reviewer will assist in checks. After data is extracted, the data will be thematically analyzed with the use of Atlas.Ti computer software. No ethics approval is required and the review will be published in peer reviewed journals and submitted to conferences.

**PROSPERO registration number:** CRD42023396459.

## Introduction

In South Africa, it is estimated that approximately 320,000 adolescents living with HIV (ALHIV) will transition from pediatric to adult antiretroviral therapy (ART) care by 2028 [1, 2]. ALHIV are expected to undergo transfer from pediatric to adult HIV care around the ages of 10–13 in South Africa [3, 4]. This transfer, known as transition, involves the planned movement from a pediatric clinic or facility to a general, adult facility where all people are seen by the same staff at the same time [5]. This transition process can be conceptualized as a shift from caregiver-led treatment to autonomous treatment-taking and care [6]. Maturo et al. [7] understand transition as the purposeful and planned transition of adolescents with chronic medical conditions from child-centered to adult-centered healthcare. Wiener et al. [8] explain that adult-centered healthcare is characterized by being independently monitored, whereas in pediatric settings the care is interdisciplinary and involves a closer relationship with the pediatric healthcare providers.

Pediatric patients living with HIV are surviving and living longer lives as a result of the implementation of ART [9]. As they are living into adulthood with the condition, consistent lifelong treatment is required [9]. Adolescents and young adults living with HIV are the fastest growing groups within the HIV population [10]. The adolescent period- ages 10–19 years (as defined by the World Health Organization)- is accompanied by barriers that challenge the transition process, and bedevil persistent adherence to ART and engagement in care. Adolescents experience marked changes, including physical, cognitive and emotional. as they develop [8, 9]. The transition process involves ALHIV taking charge of their own health and disease management which raises challenges for their engagement in care [10]. Wiener et al. [8] discuss that ALHIV who do not maintain adequate levels of adherence to ART, may experience therapeutic failure and deteriorated health, and this occur more readily when they transition prematurely–i.e., before they are ready or not adequately prepared or guided in the process. This means that they are required to presume self-responsibility for their health condition, resulting in failure of their care [8]. To achieve readiness to transition, ALHIV require a proactive and developmental approach particularly as adolescence is characterized by pervasive changes. It is well-documented that successful transition improves health outcomes and treatment skills among adolescents [11].

ALHIV have the lowest rates of retention and adherence, increasing the risk of morbidity and mortality [12]. Barriers impacting this include changes in treatment providers, treatment impositions with school, increasing responsibilities, stigma related to disclosure of HIV status, and lack of supervision [2]. Provider-related and system-related barriers include a lack of knowledge about transitioning, a lack of adolescent-centered transition protocols and a lack of implementation of adolescent-friendly services [13]. It is imperative to ensure ALHIV remain in care up into adulthood. Thus, there is a need to understand the transition to the adult care process and its outcomes and the quality of transition processes and services [14].

ALHIV in low- and middle-income countries are at risk of developmental, psychosocial, and comorbidity issues, making them a particularly vulnerable group [10]. In their review of the specific needs and challenges adolescents and young adults living with HIV transitioning to adult care face in Sub-Saharan Africa, Dahourou et al. [10] found that there are very limited data on ALHIV transitioning and their outcomes which reflects that transition of care is not a developed component of care in middle to low resource settings. Managing transition becomes particularly challenging in low-resource contexts as their healthcare systems are not adapted to the specific structural needs and the needs of ALHIV it requires to facilitate successful transition. Kung et al. [15] found that in South Africa, a middle-income country with huge discrepancies between low and high resourced settings, a lack of structure and ineffective communication between pediatric and adult health care providers is a major barrier.

Moreover, as mentioned previously, the change to adult care can be experienced as intimidating, and this can lead to the adolescent being non-adherent to their medication and clinic visits [7]. The existing barriers to successful transition results in treatment delay and lack of retention in healthcare, which inadvertently affects a worsening of immunological recovery, unsuppressed viral load, decreased quality of life in adolescents, and increased mortality [3]. A further consequence of ineffective transition leads not only to therapeutic failure and deteriorating health but can also result in multi-drug resistant HIV which can be transmitted to others [8]. Ineffective transition does not only have a negative impact on physical health, but also on the psychosocial factors that come with being diagnosed with HIV [16].

There is a need to garner an understanding of existing transition practices which address the specific needs of adolescents and is optimized to their requirements and available resources within a low- or middle-income country context [17, 18]. In higher income countries, the transition to adult care is often specialized care, whereas in lower income regions such as in Sub-Saharan Africa, it is not specialized, demonstrating the lack of transition practices tailored to ALHIV [19]. To assist this transition process, a program that is adolescent-friendly and collaborative (with healthcare providers and caregivers) where effective communication is emphasized is necessary [6, 11]. There is also a lack of written protocols to inform evidence-based and well-established transition guidelines [20]. An investigation of existing practices will garner a deeper understanding of the specific barriers that exist for ALHIV, in low- and middle-income countries, which will ultimately improve treatment outcomes. Informed and evidence-based public health approaches should be implemented to develop effective transition protocols, particularly in low-resource, high-burden settings [20]. A qualitative evidence synthesis would therefore be beneficial as it allows a greater investigation of the existing transition experiences and the specific needs of ALHIV who are transitioning to adult HIV care.

### Objectives

The aim of this review is to explore ALHIV’s experiences with practices facilitating their transition from pediatric to adult HIV care program in low and middle-income countries. The objectives are to synthesize qualitative studies exploring how ALHIV experience transition practices and to explore the influence of transition practices on their self-reported adherence, engagement in care and mental wellness (e.g., motivation and self-efficacy).

## Methods and analysis

### Registration

This protocol is submitted to PROSPERO (CRD42023396459) and adheres to the Preferred Reporting Items for Systematic Reviews and Meta-Analyses (PRISMA) guidelines (See S1 Checklist. PRISMA Checklist) [21].

### Information sources

After developing the search terms, the following databases will be utilized for this review: PubMed, Wiley Library Online, EbscoHost (PsychARTICLES, MEDLINE, Scopus, CINAHL). We will also make use of grey literature such as the WHO database to identify possible studies. To complete the search process, reference mining of included studies will be done as well as a search of Google Scholar.

### Search strategy

Prior to commencing the searches, the reviewers will consult an information specialist to determine the most effective search string. The proposed search strings for this review can be seen in Box 1:

**Box 1. Proposed search string**

(“HIV” OR “AIDS”) AND

(“Adolescent” OR “adolescence” OR “young people” OR “youth” OR “young adults” OR “teen” OR “teenager”) AND

(“antiretroviral therapy” OR “highly active antiretroviral therapy”) AND

(“health care transition” OR “transitional care” OR “transition” OR “transition models” OR “transition intervention” OR “transition practices” OR “transferal” OR “transferring”) AND

(“low-income countries” OR “middle-income countries”)

### Inclusion criteria

#### Types of studies

This review will include all qualitative and mixed method studies which will facilitate a deeper understanding of the experiences of ALHIV on transitioning from pediatric to adult HIV care. The review will specifically look at studies conducted in low- and middle-income countries. The included studies must be presented in the English language. Table 1 conveys the Sample, Phenomenon of interest, Design, Evaluation, and Research type (SPIDER) tool utilized in qualitative evidence syntheses to define research questions and search terms.

**Table 1:** SPIDER framework for selection of studies.

**SPIDER**	
**Research question: What are ALHIV’s experiences with transitioning from pediatric to adult ART in low and middle-income countries?**	
S **S****ample**	Adolescents living with HIV on ART
PI **P****henomenon of** **I****nterest**	Experiences on transitioning from pediatric to adult care
D **D****esign**	All forms of qualitative and mixed method designs which include qualitative data collection, such as interviews, focus groups, and observations.
E **E****valuation**	Experiences, engagement in care, adherence, mental wellness, motivation, self-efficacy
R **R****esearch type**	Qualitative and mixed methods

#### Topic of interest

The topic of interest of this review is experiences of ALHIV on ART who are transitioning from pediatric to adult HIV care.

#### Types of participants

The review will investigate the experiences ALHIV on ART aged 10–19 who are expected to or are experiencing the transition from pediatric to adult care. We will include studies that underline the experiences of adolescents and exclude studies that solely focus on the perspectives and experiences of healthcare providers.

#### Types of interventions

Studies that focus on the transition period (the period where adolescents are changing from pediatric to adult HIV care).

#### Time frame

This review will include studies that were not published between 2010–2023 to ensure recent and relevant findings are included.

### Exclusion criteria

#### Types of studies

The review will exclude all types of reviews, purely quantitative studies, and studies where there are no outcomes relevant to the review reported.

#### Study setting

The review will exclude all studies that were conducted in high-income, high-resource countries.

#### Time frame

This review will exclude studies that were not published between 2010–2023.

#### Language

Studies not in the English language will be excluded.

### Selection of studies

Three reviewers will be involved in the stages of the selection of studies. One reviewer will work independently on the initial screening of studies and another reviewer will assist in checks. The reviewers will work as a team to resolve disagreements, and the senior reviewer will assist in final decisions in quality checks. The PRISMA diagram will be developed concurrently as screening and selection of studies occur (See S1 Fig. Flow Diagram Template).

### Sampling of studies

Purposive sampling in qualitative research syntheses allows access to information-rich cases [22]. Large amounts of studies can amount in systematic reviews; therefore, it is necessary to ensure that for qualitative reviews, a sampling strategy is in place to include accurate and quality data. This review will use criterion sampling in the inclusion of studies as it ensures that all selected studies match the specific inclusion and exclusion criteria [22]. Criterion sampling can be used to ensure that all selected studies include ALHIV, qualitative evidence on transition experiences and how it impacts adherence, retention in care, mental wellness, motivation and self-efficacy, and are based in low- and middle-income countries. Moreover, this purposeful sampling technique will maximize the probability to reach data saturation effectively [22].

### Data extraction

This review will include a data extraction form designed for the specific review specifications and will include contextual and methodological information. This includes the context, design, data collection, aims and objectives, participants, and trustworthiness of the studies. The data extraction form will be piloted prior to data extraction to ensure relevant and accurate information will be extracted. It will be presented with first order data, such as direct quotes to provide explanations about participants’ experiences of the intervention. The quotes will then be coded and analyzed into themes.

### Quality assessment

The review will use the Critical Appraisal Skills Programme (CASP) tool for qualitative studies to assess the included studies [23]. As the review will also include studies that utilized mixed methods, it will use the Mixed Methods Appraisal Tool (MMAT) to assess the qualitative data of these studies [24].

### Assessment of confidence in review findings

The review will utilize GRADE CERQual to assess the confidence in the review findings, as outlined by Lewin et al. [25]. Two reviewers will independently assess and grade the review findings and then collaboratively decide on the final grading.

### Data management, analysis, and synthesis

Included studies will be uploaded to Covidence to manage the data and complete the screening of studies. Thematic syntheses as developed by Thomas and Harden [26] to describe the findings and relevant themes will be utilized to analyze and present the data. Thematic synthesis of review findings involves organizing codes into descriptive themes which are then interpreted into analytic themes [27]. In their thematic synthesis, Thomas and Harden [26] utilized computerized technology to analyze the data. This review will use the computer application Atlas.Ti for the thematic synthesis.

## Supporting information

S1 Checklist PRISMA checklist. Reporting checklist for the protocol of a systematic review and meta-analysis. Based on the PRISMA-P guidelines.
(PDF)

S1 Fig PRISMA flow diagram template. PRISMA Flow Diagram template for reporting selected studies.
(TIF)
