# Supplementary figures and images for: Experiences of adolescents living with HIV on transitioning from pediatric to adult HIV care in low and middle-income countries: A Qualitative Evidence Synthesis Protocol
Source: PLoS One. 2024 Feb 5;19(2):e0296184. doi: 10.1371/journal.pone.0296184 (PMC10843479; doi:10.1371/journal.pone.0296184)

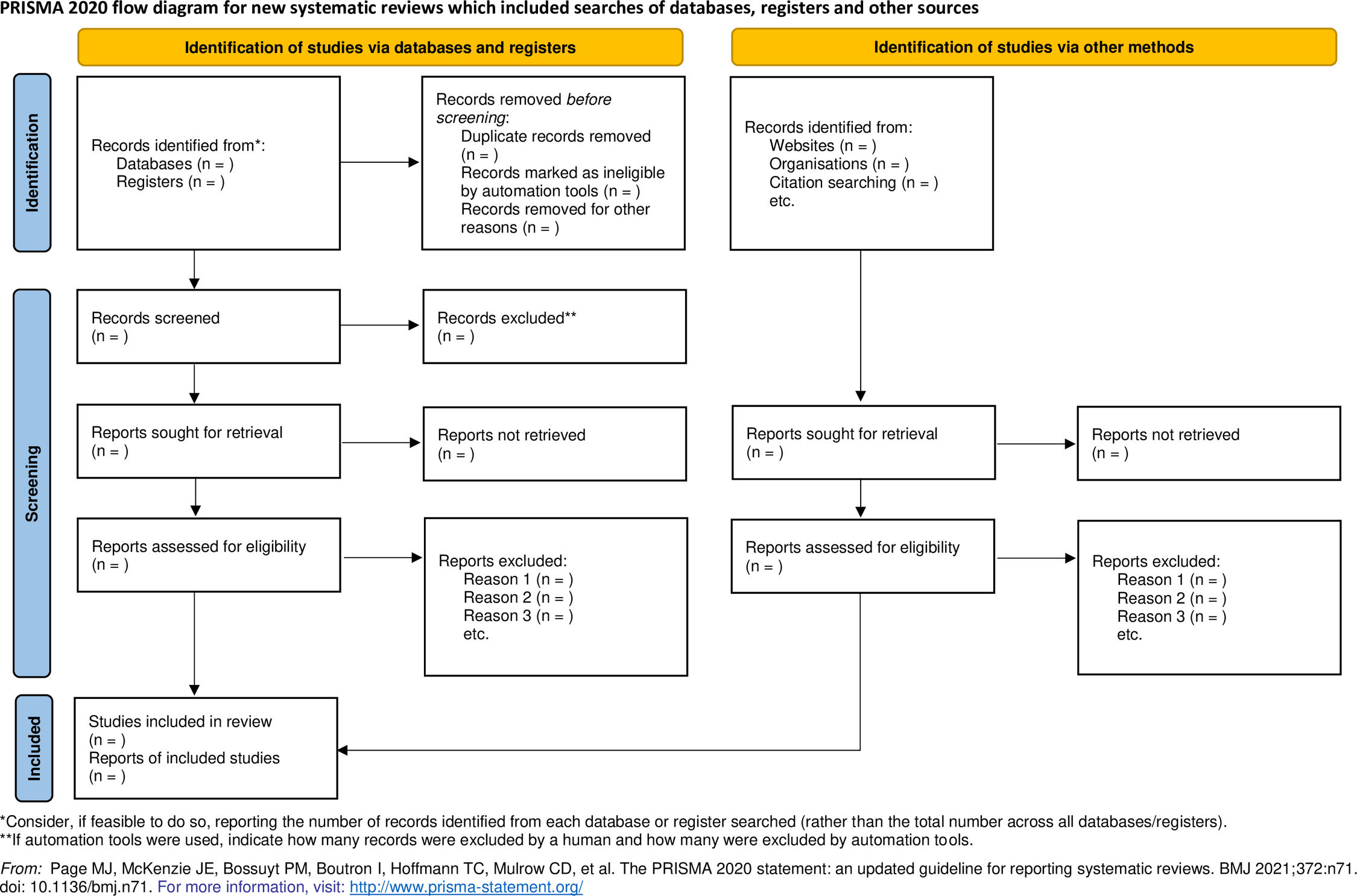

Supplement: S1 Fig — PRISMA Flow Diagram template for reporting selected studies. (TIF) [file pone.0296184.s002.tif]
